# IBD-Associated Atg16L1T300A Polymorphism Regulates Commensal Microbiota of the Intestine

**DOI:** 10.3389/fimmu.2021.772189

**Published:** 2022-01-27

**Authors:** Hongtao Liu, Ping Gao, Baoqian Jia, Na Lu, Baoli Zhu, Fuping Zhang

**Affiliations:** ^1^ CAS Key Laboratory of Pathogenic Microbiology and Immunology, Institute of Microbiology, Chinese Academy of Sciences (CAS), Beijing, China; ^2^ College of Life Science, University of Chinese Academy of Sciences, Beijing, China; ^3^ Department of Savaid Medical School, University of Chinese Academy of Sciences, Beijing, China

**Keywords:** Atg16L1T300A, microbiota dysbiosis, goblet cells, autophagy, IBD – inflammatory bowel disease

## Abstract

The development of inflammatory bowel disease (IBD) is driven by the interaction among host genetics, microbiota, and the immune system of the entire digestive tract. Atg16L1T300A polymorphism is a genetic factor that confers increased risk for the pathogenesis of Crohn’s disease. However, the exact contributions of Atg16L1T300A to intestinal mucosal homeostasis are not well understood. Here we show that Atg16L1T300A polymorphism impacts commensal bacterial flora in the intestine under a steady state. Analysis of intestinal bacteria from Atg16L1^T300A/T300A^ mice showed that they harbored an altered microbiota in both the terminal ileum and colon compared to cohoused WT mice. Interestingly, Atg16L1^T300A/T300A^ mice harbored a significant increase in the abundance of *Tyzzerella*, *Mucispirillum*, *Ruminococcaceae*, and *Cyanobacteria* which were known associated with IBD. Moreover, *Akkermansia*, a bacterium that is mucin-associated, was reduced greatly in Atg16L1^T300A/T300A^ mice. Further analysis indicated that goblet cells of Atg16L1^T300A/T300A^ mice had diminished mucin secretion that resulted from defective autophagy. Finally, Atg16L1^T300A/T300A^ mice developed more severe inflammation in the DSS colitis model than in WT mice. These results indicate that the altered microbiota in Atg16L1^T300A/T300A^ mice might be an important factor that contributed to the risk of Atg16L1T300A carriers to Crohn’s disease and supports a multi-hit disease model involving specific gene–microbe interactions.

## Introduction

Inflammatory bowel disease (IBD), which includes Crohn’s disease (CD) and ulcerative colitis (UC), is a chronic relapsing inflammatory disease characterized by impaired intestinal homeostasis and abnormal stress response (1). Chronic gut inflammation in IBD patients is also a potent risk factor for colorectal cancer, which is the second leading cause of cancer deaths with an estimated nearly 1,360,000 new cases and 694,000 deaths per year and is increasing in incidence worldwide (2). The etiology of IBD is complex. Microbial dysbiosis, genetic susceptibility, and environmental factors have been associated with the development and progression of the disease (3), although the exact cause of CD remains largely undescribed.

Trillions of bacteria living inside the gut form microbiota. The homeostasis of the gut microbiota is essential for numerous vital host physiological processes, including digestion of dietary factors, development of the gut immune system, and colonization resistance against incoming pathogens (1, 3). Dysbiosis of the gut microbiota, including alterations in frequency, diversity, and richness of microbial populations, has been associated with IBD pathogenesis (4, 5). Patients with IBD exhibit increases in the abundance of harmful taxa, or decreases in abundance of protective taxa. Research suggested a dynamic interplay between the microbiota and disease pathophysiology (6), but how dysbiosis manifests and, more specifically, how individual species are affected by host genetics are less well understood. Furthermore, numerous studies of the microbiota dysbiosis associated with IBD development have focused on the fecal samples; however, limited information is available regarding the composition of mucosa-associated microbiota and the role these microbiotas played during the IBD development.

It is widely accepted that the combination of host genetics and intestinal dysbiosis contributes to IBD. To date, genome-wide association studies (GWAS) have identified more than 230 IBD-associated susceptibility loci, a large fraction of which is associated with microbial defense pathways (7, 8). One of the most influential genes for CD susceptibility identified so far is the genetic variants at position 300 in the autophagy gene Atg16L1, resulting in a threonine-to-alanine substitution (T300A) in the C-terminal domain (8). Atg16L1, a central adaptor required for the formation of the matured autophagosome, plays a key role in mice’s intestinal epithelium (9). Atg16L1T300A mutation introduces a caspase-3 cleavage site in Atg16L1 protein that is associated with reduced levels of autophagy flux and increased risk of developing CD (10, 11). Although much progress has been made regarding the mechanism by which Atg16L1 T300A is associated with IBD, little is known about Atg16L1 T300A on the microbiota community in the intestine.

In this study, we assessed the microbiome in cohoused WT and Atg16L1^T300A/T300A^ mice using 16S rRNA gene sequencing and found that Atg16L1^T300A/T300A^ mice harbored an increased abundance of microbiota that was associated with IBD, while *Akkermansia muciniphila*, a beneficial microbiota, was decreased significantly. Moreover, Atg16L1^T300A/T300A^ mice developed more severe inflammation in a dextran sodium sulfate (DSS) colitis model. Thus, our findings indicated that microbiota dysbiosis in Atg16L1^T300A/T300A^ mice might be one of the important factors contributing to the increased susceptibility of IBD.

## Materials and Methods

### Mice

Atg16L1^T300A/T300A^ (Atg16L1^f/f^ or T300A KI) mice on a C57BL/6J background have been described previously (10). Atg16L1^f/f^ ERT2Cre^+^ mice were generated by crossed Atg16L1^f/f^ mice with ERT2Cre mice, which were treated with tamoxifen every other day for 4 times to activate ERT2Cre, and Atg16L1 was deleted to generate Atg16L1 KO mice. All mice within one experiment were cohoused for 4–5 weeks after weaning in the third week of birth to minimize differences between each line (12). All mice were bred and maintained in the SPF barrier at the Institute of Microbiology, Chinese Academy of Sciences (IMCAS). All animal studies were performed according to approved protocols from the IMCAS of Medicine Institutional Animal Care and Use Committee. The genotypes of all mice were genotyped with the following genotyping primers:

Atg16L1^T300A/T300A^:

5′-CCTGGAGCTGGGCAGTCAGGTTGGGCTCCATG-3′

5′-GCTGCTTCCCTGTCAGTCAACTGTG-3′

ERT2^Cre^:

5′-AAAGTCGCTCTGAGTTGTTAT-3′

5′-GGAGCGGGAGAAATGGATATG-3′

5′-CCTGATCCTGGCAATTTCG-3′

### DSS-Induced Colitis and Experimental Design

Cohoused, male and female, 7–8-week-old Atg16L1^T300A/T300A^ mice and their littermate control mice were used. For mild experimental colitis induction, mice were administered with 1.5% DSS (M.W. = 36,000–50,000 Da; MP Biomedicals) in their drinking water for 7 days, followed by regular drinking water for 3 days. Body weight was monitored, with changes in body weight calculated relative to initial body weight. Postmortem whole colons were harvested by blunt dissection, and colon length was measured.

### Histological Staining Analysis

Distal colon segments in the same defined region were collected carefully and were transferred immediately to 4% phosphate-buffered formaldehyde (4% PFA) solution for 24 h and embedded in paraffin. Sections of 5 μm were stained with hematoxylin and eosin (H&E) and then scanned with a light microscope (Zeiss Axio Imager A2) for further histopathological analysis. At least three slides were randomly selected and observed by a blinded pathologist. The tissue damage of each colon was scored based on the degree of epithelial damage and inflammatory infiltrate in the mucosa, submucosa, and muscularis/serosa, as previously described (13).

### Whole-Mount Immunofluorescence Staining

The isolated small intestine or colon was opened longitudinally after the adipose tissues were stripped off. Using a scissor, the intestine was cut into 2–3-cm equal sections. Each section was placed onto a wax plate, and each end was pinned down so the section was slightly stretched with the mesenteric line uppermost; any excess mesentery was trimmed. Tissues were fixed in 4% PFA for 1 h before being rinsed three times for 5 min in PBS. The tissues were flooded with fluorescein isothiocyanate–labeled *H. pomatia lectin* (Invitrogen, L11271) for 2 h at room temperature. Tissues were mounted on glass slides and examined under a Zeiss Axio Imager A2 fluorescence microscope with a ×10 objective.

### Fluorescence *In Situ* Hybridization

Colon tissues were prepared for fluorescence *in situ* hybridization (FISH), analysis as previously described (14). Briefly, after mouse intestines were fixed in 4% PFA, and after deparaffinization and rehydration in hybridization buffer (0.9 M NaCl, 0.1% SDS, and 20 mM Tris–HCl, pH 7.4), the tissues were incubated 2 hours at 50°C in the dark with Alexa 532-conjugated Eubacteria EUB338 (5′-GCTGCCTCCCGTAGGAGT-3′) or non-EUB338 probe for bacterial 16S rRNA genes (15). The sections were then washed three times with a hybridization solution for 15 min, counterstained with DAPI, and mounted. The sections were imaged with an inverted microscope (Leica SP8) using a Leica ×10 objective. Distances between intestinal epithelial cells and luminal bacteria were quantified by using the ImageJ tool.

### PAS Staining

Mouse distal ileum or colon samples were fixed in 4% PFA in PBS for 24 h, dehydrated, and embedded in paraffin. Tissue slices (5 μm in thickness) were mounted on positively charged glass and dewaxed.

For periodic acid–Schiff (PAS) staining, sections (5 μm) were cut and stained with PAS. Briefly, the slides were incubated for 5 min in 0.5% periodic acid solution, washed with ddH_2_O, and then incubated for 20 min in Schiff reagent, washed 5 min in tap water. The slides were then counterstained with hematoxylin for 1 min, after which the slides were dehydrated, mounted, and observed with a light microscope (Zeiss Axio Imager A2).

### Transmission Electron Microscopy

Sections of mouse intestines from euthanized mice were washed with ice-cold PBS and cut into pieces about 3 mm in length. Selected tissues were fixed in 2.5% glutaraldehyde and 2% paraformaldehyde in 0.1 M sodium phosphate buffer (PB buffer, pH 7.4) at 4°C overnight. Then, samples were rinsed 5–7 times in PB buffer, postfixed in 1% osmic acid for 2 h, and washed 5–7 times in PB buffer. Dehydration was done with an acetone gradient followed by infiltration with Epon 812 resin and baked overnight at 60°C. Hardened blocks were cut using a Leica Ultracut Ultramicrotome (Leica EM UC6) and stained using 2% uranyl acetate and lead citrate. Images were obtained using a transmission electron microscope (JEM-1400) at 80 kV. Goblet cells were measured from five animals for each genotype.

### Isolation of Intestinal Epithelial Cells

Epithelial cells were obtained from freshly isolated colons and flushed from both ends with sterile PBS. Colons were then opened longitudinally and washed with PBS. Mucus was removed by gently scraping the villus with a cover glass slide. Intestines were washed and incubated in 10 ml PBS containing 30 mM EDTA and 1.5 mM DTT on ice for 20 min (16). Intestines were then removed and briefly washed in PBS and incubated in 10 ml PBS containing 30 mM EDTA at 37°C at 220 RPM for 10 min. The cells were subjected to 30-s vigorous shaking, then centrifuged at 1,000g for 5 min at 4°C, washed in PBS, and then resuspended in PBS buffer. Cell pellets constituting isolated intestinal epithelial cell fractions were lysed for Western blot to detect Atg16L1 and LC3.

### Immunoblot

Colonic epithelium cells were isolated as described above (16). Isolated cells were lysed in RIPA buffer with protease inhibitors and subjected to SDS-PAGE and transferred to polyvinylidene difluoride (PVDF) membranes. Then, PVDF membranes were blocked by exposure to PBST containing 5% non-fat dry milk for 1 h at room temperature. The blocked membranes were probed by incubation with the indicated antibody at 4°C overnight. Proteins were detected with HRP-conjugated secondary antibody and visualized by ECL (GE). The following antibodies were used: anti-Atg16L1 (MBL, M150-3), anti-LC3 (Novus Biologicals, NB100-2331), and anti-actin (Santa Cruz Biotechnology, sc-8432).

### RNA Isolation and Gene Expression Analysis

RNA was extracted from isolated colonic epitheliums with TRIzol Reagent (Invitrogen) following the manufacturer’s instructions. cDNA was generated using a High-Capacity cDNA Reverse Transcription Kit (Applied Biosystems). Real-time PCR was performed using the SYBR Green Master Mix (Applied Biosystems) on an ABI 7500 thermal cycler in duplicate. Gene-expression values were calculated by using 2^-△△Ct^ normalized to HPRT. Primers for quantitative PCR were used as follows:

Mouse–Muc2-F: ACGATGCCTACACCAAGGTC

Mouse–Muc2-R: TGATCTTCTGCATGTTCCCA

Mouse HPRT-F: GTCCCAGCGTCGTGATTAGC

Mouse HPRT-R: TGGCCTCCCATCTCCTTCA

### 16S rRNA Gene Sequencing

Stool samples for 16S rRNA amplicon surveys were collected into a 2-ml sterile tube and stored at -80°C prior to DNA extraction. Mucus scraping samples were collected as previously described (17). Briefly, following sacrifice of mice, the distal ileum and whole colon were excised and filleted open. Large fecal matter was gently removed if present, and a brushing of the colonic mucosal surface was performed to test for the mucosal-associated microbiome. Then, the collected fecal pellets and mucus scrapings were processed for DNA isolation according to the recommended manual (Qiagen). PCR amplification, library preparation, library-quality inspection, and quantification were performed, and the set TAG sequence was used to distinguish the samples. 16S rRNA sequencing of the V4 region was performed on an Illumina HiSeq 2500 high-throughput sequencing platform to sequence the qualified libraries.

### Statistical Analysis

Data are expressed as mean ± SEM. Two groups were compared by using a non-parametric test analysis of variance followed by a Mann–Whitney test analysis. Multiple-comparison analyses were performed using one/two-way ANOVA followed by Tukey or Bonferroni *post hoc* tests using GraphPad Prism 7 (GraphPad Software Inc.). p < 0.05 was considered as indicating a statistically significant difference.

## Results

### Atg16L1^T300A/T300A^ Mice Display a Slightly Different Microbial Composition in Fecal at a Steady State

**Figure 1:**
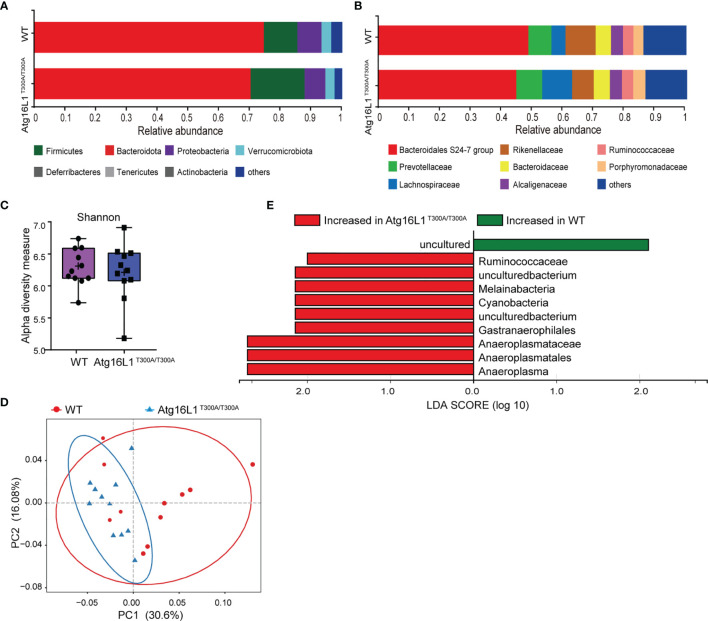
Atg16L1T300A mice display a slightly different microbial composition in fecal at a steady state. WT and Atg16L1^T300A/T300A^ mice were cohoused in a specific-pathogen-free (SPF) facility for 4–5 weeks after weaning at the third week of birth. (WT mice n = 11 and Atg16L1^T300A/T300A^ mice n = 11). Stool samples were collected and analyzed by 16S rRNA gene amplicon sequencing. **(A, B)** The relative abundance of dominant bacteria at phyla **(A)** and families **(B)** level microbiota composition determined by 16S rRNA gene amplicon sequencing of stool samples from SPF cohoused WT vs. Atg16L1^T300A/T300A^ mice at steady state. Colored bars represent the relative abundance of particular taxa averaged in each group. **(C)** Box-and-whisker plot (boxes show median, whisker denotes minimum to maximum range) of microbiota alpha-diversity within the fecal microbiomes of WT vs. Atg16L1^T300A/T300A^ SPF mouse samples calculated using the Shannon index. **(D)** PCoA plot of the fecal microbiota composition (unweighted UniFrac distances). **(E)** Histogram of LDA value distribution identified by LEfSe analysis. Each symbol represents an individual animal (non-parametric test followed by a Mann–Whitney test).

Previous studies have shown that IBD development is associated with dysbiosis (2). Atg16L1 is an autophagy gene involved in the handling of intracellular bacteria. Cells or mice bearing Atg16L1 T300A polymorphism have a reduced capacity of bacterial clearance (10, 18). Although it has been reported that the Atg16L1 T300A mutation is associated with alterations in gut microbiota, there is a lack of further detailed investigation, and the factors that contributed to altered gut microbiota have never been studied. Thus, to determine the effect of Atg16L1 T300A on commensal bacteria at a steady state, we performed experiments by using cohoused WT and Atg16L1^T300A/T300A^ mice to reduce the influence of microbiome heritability as a confounding factor. Because fecal samples are widely used in mouse studies to survey the microbiome and its relationship to IBD, we first performed *16S* rRNA gene sequencing on feces of WT and Atg16L1^T300A/T300A^ mice to determine the composition and diversity of the fecal associated microbiota. We found that the taxonomy-based analysis did not have broad alterations in the phylum and family composition of commensal bacteria between Atg16L1^T300A/T300A^ and WT mice (**Figures 1A, B** **)**, and alpha-diversity analyses based on the Shannon index and Bray–Curtis distance also showed no significant differences between Atg16L1^T300A/T300A^ mice and WT controls (**Figure 1C**). Next, we performed unweighted UniFrac PCoA analysis to investigate the differences in species complexity and structural alterations in gut microbial communities (β-diversity), and we found a minor difference between the WT and Atg16L1^T300A/T300A^ microbiotas (**Figure 1D**). Moreover, we applied the linear discriminant analysis (LDA) effect size (LEfSe) method for high-dimensional biomarker discovery to interpret the microbiota between Atg16L1^T300A/T300A^ and WT commensal composition. We found nine differentially abundant taxonomic clades with an LDA score higher than 2.0, indicating that these bacteria significantly differ in WT and Atg16L1^T300A/T300A^ mouse feces. Among the nine differentially abundant taxonomic clades, the opportunistic pathogens *Ruminococcaceae* and *Cyanobacteria* were enriched in Atg16L1^T300A/T300A^ mice (**Figure 1E**) and have been reported to be increased in fecal samples from the mouse model of ulcerative colitis (19, 20) and in human patients of Crohn’s disease (21). Thus, this finding indicated that the Atg16L1 T300A variant slightly influenced the microbial composition in fecal samples at a steady state.

### Alteration of Microbial Communities in the Mucosa of Atg16L1^T300A/T300A^ Mice

**Figure 2:**
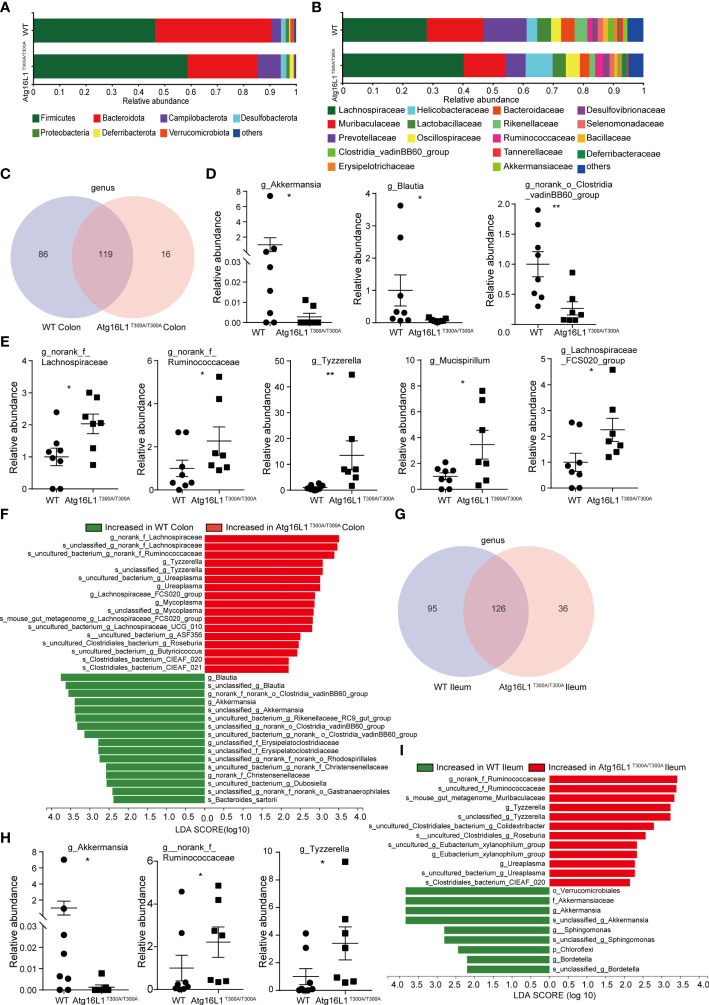
Colonic and distal ileum mucosa microbial communities are altered in Atg16L1^T300A/T300A^ mice. WT and Atg16L1^T300A/T300A^ mice were cohoused in the SPF facility for 4–5 weeks after weaning at the third week of birth (WT mice n = 8 and Atg16L1^T300A/T300A^ mice n = 7). Mice sacrificed after being cohoused for 4–5 weeks and whole colon and distal ileum excised. DNA was extracted from the colonic mucosa lining, and 16S rRNA gene sequencing was performed to profile bacterial taxa in each group. **(A, B)** The relative abundance of dominant bacteria at phyla- **(A)** and family- **(B)** level microbiota composition in colonic mucosa. Colored bars represent the relative abundance of particular taxa averaged in each group. **(C)** Venn diagram showing unique and shared genus among the WT and Atg16L1^T300A/T300A^ mouse colon mucosa. **(D)** Relative abundance of *Akkermansia*, *Blautia*, and norank_o_*Clostridia_vadinBB60*_group genus in WT and Atg16L1^T300A/T300A^ mouse colon mucosa scraping samples. Each dot represents an individual mouse sample. **(E)** Relative abundance of norank_f_*Lachnospiraceae*, norank_f_*Ruminococcaceae*, *Tyzzerella*, *Mucispirillum*, and *Lachnospiraceae_FCS020*_group genus in WT and Atg16L1^T300A/T300A^ mouse colon mucosa scraping samples. Each dot represents an individual mouse sample. **(F)** Histogram of LDA value distribution identified by LEfSe analysis of colon mucosa scraping samples. **(G)** The common and specific genus was shown by the Venn diagram between WT and Atg16L1^T300A/T300A^ mouse ileal mucosa. **(H)** Relative abundance of *Akkermansia*, norank_f_*Ruminococcaceae*, and *Tyzzerella* genus in WT and Atg16L1^T300A/T300A^ mouse ileum mucosa scraping samples. Each dot represents an individual mouse sample. **(I)** Histogram of LDA value distribution identified by LEfSe analysis of ileum mucosa scraping samples. The relative bacterial abundance was calculated by normalizing each WT and T300A mouse bacterial abundance with the mean value of bacterial abundance of all WT mice. *p < 0.05, **p < 0.01 (non-parametric test followed by a Mann–Whitney test).

While fecal samples are the predominant material used for microbial community analysis, it may not be the ideal sample for analysis of the gut microbiome. Because IBD is characterized by a compromised mucosal barrier and inappropriate immune activation by commensals mislocalized to the mucosa (22), and bacteria reside in the colonic mucus gel layer, and the colonic mucus microbiome was more closely correlated with colitis severity than that of the alterations in the fecal microbiomes (23). Thus, investigating the microbial communities of the mucus layer may provide more important information that is key to understanding the mechanisms by which the Atg16L1 T300A variant affected CD development. In the next study, we investigated the interrelationship between the Atg16L1 T300A variant and the composition of microbiota in the ileum and colon mucosa of Atg16L1^T300A/T300A^ and WT mice. For this end, colons and terminal ileum were isolated from WT and Atg16L1^T300A/T300A^ mice, and the content was removed. Then, the mucosa scraping sample was collected and used to perform 16S rRNA gene amplicon surveys to detect associated mucosal bacteria. The UniFrac PCoA analysis of microbial communities in the mucosa indicated that the overall microbiome diversity is comparable between WT and Atg16L1^T300A/T300A^ mice (**Figure S1A**). Moreover, the relative taxa abundance is different in colon mucosa between the WT and Atg16L1^T300A/T300A^ mice. The most abundant taxa at the phylum and family levels in the colon are shown in (**Figures 2A, B** **)**. At the phylum level, the abundance of *Firmicutes*, *Campilobacterota*, and *Deferribaterota* was amplified, while *Bacteroidetes* was declined in Atg16L1^T300A/T300A^ mice compared to that of the WT group. This observation was consistent with the reports that decreased abundance of Bacteroidetes can contribute to disease pathogenesis in IBD (24, 25). In addition, we also found that the levels of *Verrucomicrobiota* were reduced in Atg16L1^T300A/T300A^ mouse colonic mucosa, and a similar observation has been reported in IBD patients (26, 27).

At the family level, the relative abundance of *Helicobacteraceae*, *Ruminococcaceae*, and *Deferribacteraceae* in the Atg16L1^T300A/T300A^ mice was increased compared with that of the WT mice (**Figure 2B**). *Helicobacteraceae* played a pathogenic role in the development of CD in a considerable proportion of children (28, 29), and *Ruminococcaceae* and *Deferribacteraceae* were increased in DSS-treated colitis mice (30). Interestingly, the relative abundances of *Akkermansiaceae* in Atg16L1^T300A/T300A^ mice were decreased dramatically compared with those of WT mice. *Akkermansia muciniphila* (*A. muciniphila*), a mucin-degrading bacterium, has been proved associated with healthy mucosa and plays a protective role in regulating intestinal epithelium and mucosal function (31). *A. muciniphila* reduction has been shown in ulcerative colitis (UC) and Crohn’s disease (CD), both in clinically active diseases and during the remission phase. Moreover, it has been reported that *A. muciniphila* can act as a biomarker to predict CD (31, 32). Moreover, a study in 2011 demonstrated an inverse correlation between *A. muciniphila* levels and the severity of acute appendicitis (33).

Venn diagrams are extensively used for the visualization of relationships between different group datasets to illustrate their similarities and differences. At the genus level, Venn diagram analysis indicated that the T300A variant led to reduced microbiota diversity compared with WT control mice (**Figure 2C**) (86 and 16), suggesting that Atg16L1 T300A polymorphism reduced the mucosa-associated bacterial composition. In addition, Wilcoxon rank-sum test analysis based on genus level found that several taxonomic clades significantly differed between Atg16L1^T300A/T300A^ mice and those of cohoused WT controls. For instance, the proportions and relative abundance of *Akkermansia*, *Blautia*, and norank_o_*Clostridia_vadinBB60*_group diminished significantly in the colon of Atg16L1^T300A/T300A^ mice (**Figure 2D** and **Figure S1B**), while the opportunistic pathogens such as norank*_*f*_Lachnospiraceae*, norank*_*f*_Ruminococcaceae*, *Tyzzerella*, *Mucispirillum*, and *Lachnospiraceae_FCS020_*group significantly increased in Atg16L1^T300A/T300A^ mice (**Figure 2E** and **Figure S1B**), and the changes of the above bacteria were associated with IBD development (34–37).

To gain more insight into the changes of microbiota resident in colon mucus in Atg16L1^T300A/T300A^ mice, we next analyzed the microbiota abundance from the genus to species levels by using the LEfSe method as a visualization tool. We found more than twenty differentially abundant taxonomic clades with an LDA score higher than 2.0 in Atg16L1^T300A/T300A^ mice. Many of them were consistent with Wilcoxon rank-sum test analysis (**Figure 2F**) and had been reported associated with IBD patients (26, 31). These results indicated altered IBD-associated microbiota resident in Atg16L1^T300A/T300A^ mice even under a steady state.

We next analyzed the microbiota composition in ileum mucosa. We found that the T300A risk allele altered the composition of mucosa-associated bacteria in Atg16L1^T300A/T300A^ mice and was similar to what was observed in the colon mucus. A Venn diagram analysis indicated that Atg16L1T300A led to a reduction in the abundance of genus compared with WT mice (95 and 36) (**Figure 2G**), suggesting that Atg16L1 T300A polymorphism reduced the mucosa-associated bacterial diversity in the ileum as well. Wilcoxon rank-sum test analysis indicated that the proportions and relative abundance of probiotic bacteria *Akkermansia* decreased dramatically in the ileum, and the relative abundance of opportunistic pathogens *Tyzzerella* and norank*_*f*_Ruminococcaceae*, profoundly overrepresented bacteria in IBD, were also increased in Atg16L1^T300A/T300A^ mouse ileum mucosa (**Figure 2H** and **Figure S1C**). LEfSe analysis from genus to species further confirmed the finding that the T300A risk allele significantly altered the abundance of bacteria residing in the mucus gel layer of the ileum, and approximately twenty differentially abundant taxonomic clades with LDA score higher than 2.0 in Atg16L1^T300A/T300A^ mouse ileum mucosa were found (**Figure 2I**).

Collectively, these findings indicated that the mucus-associated gut microbiome composition in the ileum and colon of Atg16L1^T300A/T300A^ mice displayed an IBD-associated microbe species even under the steady state in our facility.

### Atg16L1^T300A/T300A^ Mice Exhibit Goblet Cell Morphology Abnormality in the Intestine

**Figure 3:**
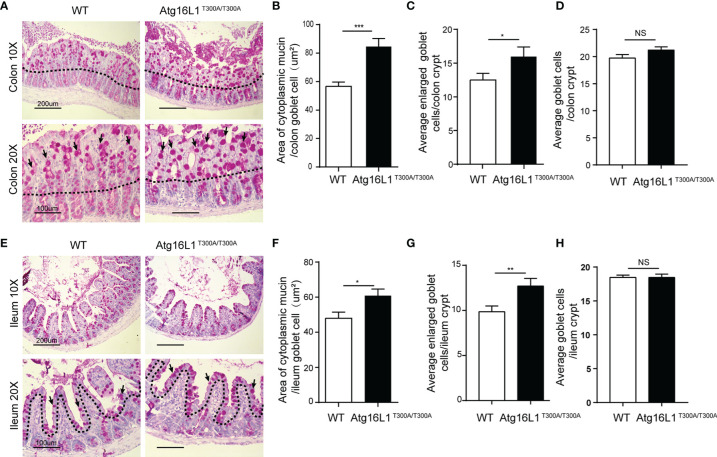
Atg16L1^T300A/T300A^ mice display morphological abnormalities in their Goblet cells. **(A)** PAS-stained colonic sections of cohoused WT and Atg16L1^T300A/T300A^ mice. Hematoxylin is in blue. Black dotted lines outline the surface epithelium. Arrows indicate surface goblet cells (scale bars: down, 100 μm and upper, 200 μm). **(B)** Quantification of average mucin area/colon goblet cells, and **(C)** average number of enlarged goblet cells in the upper crypt region of cohoused WT and Atg16L1^T300A/T300A^ mouse colon. **(D)** Quantification of the total number of goblet cells per crypt in the colon of WT and Atg16L1^T300A/T300A^ mice (n = 6–8 mice/group from 3 independent experiments; 50 goblet cells measured/mouse). This analysis was performed on upper crypt goblet cells [above the upper dashed line in **(A)**]. **(E)** PAS-stained ileum sections of cohoused WT and Atg16L1^T300A/T300A^ mice. Hematoxylin is in blue. Black dotted lines outline the surface epithelium. Arrows indicate surface goblet cells (scale bars: down, 100 μm and upper, 200 μm). **(F)** Quantification of the average area of cytoplasmic mucin/ileum goblet cells in the upper crypt region of WT and Atg16L1^T300A/T300A^ mouse ileum. **(G)** Quantification of the average number of enlarged goblet cells in the upper crypt region and **(H)** the average total number of goblet cells per crypt in the ileum of WT and Atg16L1^T300A/T300A^ mice (n = 6–8 mice/group from 3 independent experiments; 50 goblet cells measured/mouse). This analysis was performed on upper crypt goblet cells [above the upper dashed line in **(F)**]. Data are expressed as mean and s.e.m. *p < 0.05, **p < 0.01, ***p < 0.001, NS, not significant (non-parametric test followed by a Mann–Whitney test).

The data above showed that *Akkermansia* was decreased dramatically in the mucus of Atg16L1^T300A/T300A^ mice in both colon and ileum mucosa. *Akkermans*ia, as a gram-negative and strictly anaerobic bacterium, tended to widely colonize the nutrient-rich intestinal mucus gel layer (MGL) and was particularly effective in increasing mucus thickness and gut barrier function to maintain the homeostasis of the intestinal microbiota (38). Colonization with *Akkermansia* has been reported to promote mucosal wound healing (39, 40). Its metabolic and mucolytic activity makes *Akkermansia* a key species in the mucus layer, stimulating beneficial mucosal microbial networks. Because the MGL is composed mainly of mucin (Muc2) secreted by goblet cells, we hypothesized that the decreased *Akkermansia* composition in the Atg16L1^T300A/T300A^ ileum and colon mucosal layer may be correlated with the defect of the mucin secretion (41–43). To test this possibility, we first investigate the morphology of the goblet cell in the colon. Colon sections from Atg16L1^T300A/T300A^ and cohoused WT mice were performed with PAS staining. PAS staining labels highly glycosylated proteins, most notably in goblet cell mucins. We found that Atg16L1^T300A/T300A^ mice had larger areas of mucin (**Figures 3A, B** **)** and enlarged goblet cells within the surface epithelial cuffs than WT controls (**Figure 3C**). However, the total number of goblet cells per crypt did not change compared to WT controls (**Figure 3D**), suggesting that the Atg16L1 T300A variant did not regulate the differentiation of goblet cells in the gut. These results are similar to what was observed in mice with a complete absence of autophagy protein Atg5 (44).

Similarly, although the total number of goblet cells per crypt was comparable in the ileum between WT and Atg16L1^T300A/T300A^ mice (**Figures 3E, H** **)**, the ileum goblet cells of Atg16L1^T300A/T300A^ mice also contained larger areas of cytoplasmic mucin and an increased number of enlarged goblet cells (**Figures 3F, G** **)**, which is in contrast to the previous report that the enlarged goblet cell number in the small intestinal was unchanged (45). The discrepancy between the two studies was probably due to the difference in the microbiota of the two-mouse facility.

### Secretion Capacity of Goblet Cells Was Attenuated in Atg16L1^T300A/T300A^ Mice

**Figure 4:**
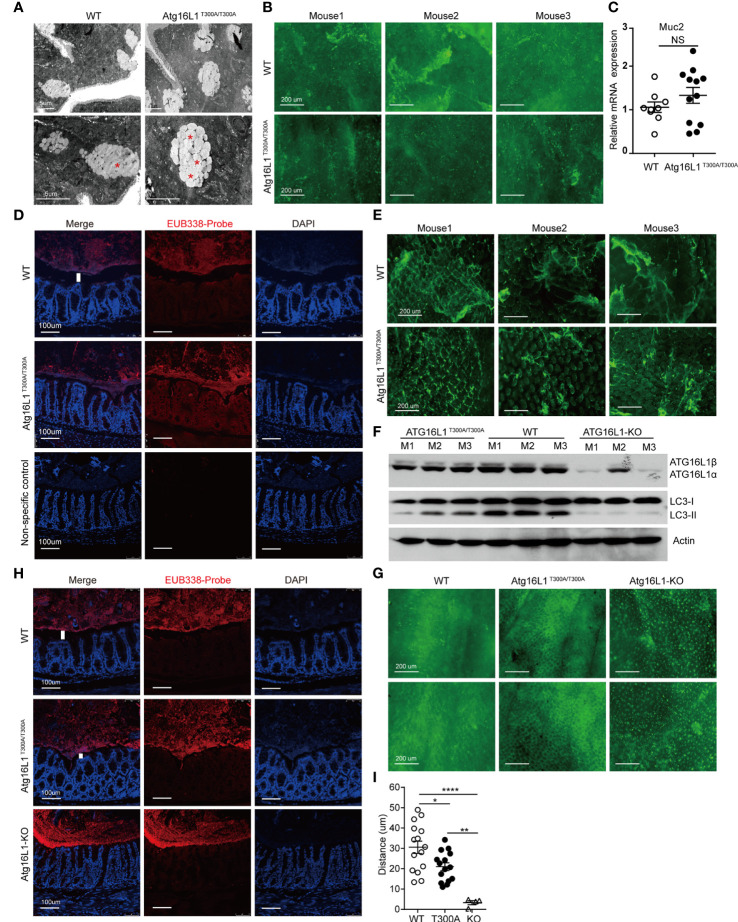
Impaired function of mucin secretion in Atg16L1^T300A/T300A^ goblet cells is autophagy-dependent. Isolated colon or distal ileum tissues from cohoused WT and Atg16L1^T300A/T300A^ mice stained with Helix pomatia lectin for mucus secretion by whole-mount staining, and microbiota localization in the colon was detected by FISH staining. n= 6–8 mice/group. **(A)** Representative transmission electron microscopy images were taken from intestine sections of cohoused WT and Atg16L1^T300A/T300A^ mice. Red asterisks indicate enlarged granules; n = 5 mice per genotypes. Scale bars, 5 μm. **(B)** Representative whole-mount images of tissues taken from the colon mucosal surface of cohoused WT and Atg16L1^T300A/T300A^ mice stained with Helix pomatia lectin (Lectin-HPA) that labels mucus (green). Images are representative of at least three independent experiments. Scale bars, 200 μm. **(C)** Quantitative real-time PCR analysis of mucus (Muc2) mRNA of cohoused WT and Atg16L1^T300A/T300A^ mouse colonic epithelium; results are presented relative to those of wild-type mice. **(D)** Visualization of microbiota localization relative to the intestinal mucosa of the colon surface by FISH. Sections were hybridized with the EUB338 probe recognizing the 16S rRNA genes of all bacteria (red) or non-EUB338 as a non-specific control and counterstained with DAPI (blue). Scale bar: 100 μm. **(E)** Representative whole-mount images taken from the ileum mucosal surface in cohoused WT and Atg16L1^T300A/T300A^ mice stained with Helix pomatia lectin (Lectin-HPA) that labels mucus (green). **(F)** Colonic epithelial cells were isolated from the colon of cohoused WT, Atg16L1^T300A/T300A^, and Atg16L1-KO mice, and Western blot analysis was used to detect Atg16L1 and LC3 changes from whole-cell lysates. Actin was used as loading control. n = 3 mice/group. **(G)** Representative whole-mount images of tissues taken from the colon mucosal surface of cohoused wild-type, Atg16L1^T300A/T300A^, and Atg16L-KO mice stained with Helix pomatia lectin (Lectin-HPA) that labels mucus (green). **(H)** Visualization of microbiota localization relative to the intestinal mucosal of the colon surface by FISH. Sections were hybridized with the EUB338 probe recognizing the 16S rRNA genes of all bacteria (red) and counterstained with DAPI (blue). Scale bar: 100 μm. The white column indicates the distance between the apical surface of the epithelium and the luminal contents. **(I)** Quantification of the distance of separating villi from microbiota in **(D, H)**. Data are from at least three independent experiments. Each symbol represents an individual animal. *p < 0.05, **p < 0.01, ****p < 0.0001. NS, not significant (non-parametric test followed by a Mann–Whitney test; one/two-way ANOVA analysis followed by a Tukey/Bonferroni *post hoc* test for three-group analysis).

Goblet cells are a secretory lineage that specializes in mucus production. Goblet cells secrete mucus, which forms a layer that overlays the epithelium and forms an important barrier to intestinal microbes (46). Although it has been reported that Atg16L1^T300A/T300A^ results in enlarged goblet cells within the surface epithelial cuffs (45), the consequence of enlarged goblet cells in Atg16L1^T300A/T300A^ mice has never been studied. Intestinal mucus is composed of multiple highly glycosylated proteins called mucins. Mucins are stored in secretory granules; constitutive secretion occurs by fusion of individual mucin granules with the apical plasma membrane (47). Mucin granules can accumulate within the goblet cell cytoplasm leading to increased size, which can be secondary to either increased mucin production or diminished secretion. To examine whether the enlarged goblet cell in Atg16L1^T300A/T300A^ mice was due to impaired secretion, we first used transmission electron microscopy to visualize the theca of goblet cells, which is normally packed with mucin granules. We found that in WT mice, once the theca-containing mucin granules reach the apical surface of the intestinal epithelium, they fuse with the epithelium, releasing the stored mucins and associated proteins into the intestinal lumen. In contrast, the goblet cell from Atg16L1^T300A/T300A^ mice was featured with an increased accumulation of intracellular mucin granules and an apparent inability of these granules to fuse with the apical surface of the intestinal epithelium (**Figure 4A**), which resulted in a deficiency of mucin release. To further confirm the defect of mucin secretion in Atg16L1^T300A/T300A^ mice, we detected mucin in the lumen of the intestine by whole-mount staining. To this end, colons obtained from cohoused WT and Atg16L1^T300A/T300A^ mice were collected and opened longitudinally after the adipose tissues were stripped off. 2–3-cm equal intestine sections were fixed and stained with fluorescein isothiocyanate–labeled H. pomatia lectin at room temperature, after which the images were taken immediately above the colon mucosal surface. We found that mucus secreted into the colon lumen was reduced in Atg16L1^T300A/T300A^ mice compared with that of cohoused WT controls (**Figure 4B**). These results indicate that Atg16L1^T300A/T300A^ goblet cells had a defect in granule exocytosis and a lack of intact mucus layer.

The diminished secretion of mucin in the lumen of the Atg16L1^T300A/T300A^ mice could be due to impaired mucin gene expression. Muc2 is the predominant mucin secreted by goblet cells, so we analyzed Muc2 expression in the colon epithelium from cohoused WT and Atg16L1^T300A/T300A^ mice by real-time PCR. We found no difference in the expression of Muc2 in cohoused WT and Atg16L1^T300A/T300A^ mice (**Figure 4C**), supporting the conclusion that the reduced mucin observed in the colon was not due to the gene expression but the deficiency of secretion.

The MGL lining the colon is integral to preventing bacterial invasion and maintaining intestinal homeostasis in health and disease. MGL defects allow bacteria to contact the colonic surface directly and are commonly observed in IBD (48). Having observed defected mucin secretion of goblet cells in Atg16L1^T300A/T300A^ mice, we next determined whether the reduced secretion of mucin could impair the homeostasis of mucus lining. We examined the spatial distance between mucosal surfaces and the luminal contents by fluorescence *in situ* hybridization (FISH) assay with a universal 16S ribosomal RNA (rRNA) gene probe. We found that the spatial distance of mucosal surfaces and the luminal contents were approximately ~45 μm (from the villus tip) in WT mice (**Figures 4D, I** **)**. In contrast, a marked decrease in spatial distance between mucosal surfaces and the luminal contents was observed in Atg16L1^T300A/T300A^ mice (~25 μm). We also examined mucin secretion in the ileum as above. The results showed decreased mucus secretion in the ileum lumen of Atg16L1^T300A/T300A^ mice compared with WT controls (**Figure 4E**).

Collectively, these findings indicate that Atg16L1T300A polymorphism regulated the secretion capacity of intestinal goblet cells, which may contribute to microbiota dysbiosis in Atg16L1^T300A/T300A^ mice.

### The Impaired Function of Atg16L1 T300A Goblet Cell Is Autophagy-Dependent

To further investigate the mechanism by which Atg16L1T300A polymorphism regulates goblet cell secretion, we next investigated whether the impaired secretion function of Atg16L1 T300A goblet cells is due to the defective autophagic process which is required for proper secretion of mucus granules. We crossed Atg16L1^f/f^ mice with ERT2Cre mice to generate Atg16L1^f/f^&ERT2Cre^+^ mice. Tamoxifen was orally administrated to Atg16L1^f/f^&ERT2Cre^+^ mice to generate Atg16L1-KO mice. Colon epithelial cells collected from WT, Atg16L1^T300A/T300A^, and Atg16L1-KO mice were lysed and immunoblotted with the anti-Atg16L1 antibody; we found that Atg16L1 expression was comparable between WT and Atg16L1^T300A/T300A^ mice, but almost completely lost in Atg16L1-KO mice (**Figure 4F**). These results indicated that Atg16L1 T300A mutation did not influence the Atg16L1 expression and tamoxifen administration activates ERT2Cre and deleted Atg16L1 expression. We next studied autophagy induction in the intestine epithelial cell (mainly containing goblet cell). The colon epithelial cell was collected from WT, Atg16L1^T300A/T300A^, and Atg16L1 KO mice as above, and autophagy induction was detected by LC3 conversion. We observed that LC3II conversion was impaired significantly in intestinal epithelial cells of Atg16L1^T300A/T300A^ mice compared with those of cohoused WT mice, indicating that autophagy was impaired in Atg16L1^T300A/T300A^ intestinal epithelial cells; LC3II was almost completely lost in epithelial cells of Atg16L1-KO mice (**Figure 4F**).

To detect the effect of autophagy on mucus secretion directly, we performed whole-mount staining in both Atg16L1^T300A/T300A^ and Atg16L1-KO mice as above, and the results showed that Atg16L1 KO mice had more drastic effects on mucin secretion; mucus secreted was almost completely lost in Atg16L1-KO mice compared with that of WT controls (**Figure 4G**). These findings suggested that impaired mucin secretion in Atg16L1^T300A/T300A^ mice is autophagy-dependent, which is consistent with the previous report that autophagy was required for mucin granule exocytosis of goblet cells (44). Finally, we performed FISH staining with the colon of WT, Atg16L1^T300A/T300A^, and Atg16L1-KO mice to examine the spatial distance between mucosal surfaces and the luminal contents. The result showed that the distance between the apical surface of the epithelium and the luminal contents in Atg16L1-KO mice reduced dramatically compared with that of Atg16L1^T300A/T300A^ and WT mice, and commensal bacteria were directly attached to the epithelia of Atg16L1-KO mice (**Figures 4H, I** **)**. These findings indicated that reduced mucin secretion of goblet cells in Atg16L1 T300A mice is autophagy-dependent.

### Atg16L1^T300A/T300A^ Mice Exhibit More Severe Inflammation in DSS-Induced Colitis

**Figure 5:**
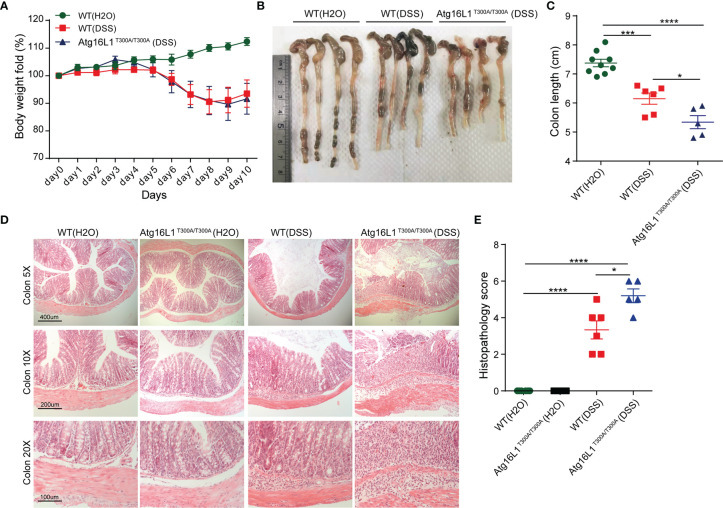
Atg16L1^T300A/T300A^ mice exhibit more severe inflammation in DSS-induced colitis. **(A)** Weight loss of WT mice with drinking water (n = 9, 3 male and 6 female) and cohoused WT (n = 6, 2 male and 4 female) and Atg16L1^T300A/T300A^ mice (n = 5, 1 male and 4 female) treated with 1.5% DSS for 7 days followed by 3 days of regular drinking water. **(B, C)** Gross pathology of colons on day 10 post 1.5% DSS. **(D)** Representative H&E staining of distal colon sections obtained from cohoused WT and Atg16L1^T300A/T300A^ mice with drinking water and cohoused WT and Atg16L1^T300A/T300A^ mice on day 10 post DSS treatment (scale bar, represents 400 μm; images acquired at 5×; represents 200 μm; images acquired at 10×; represents 100 μm; images acquired at 20×). **(E)** Histological analysis of distal colon harvested at day 10 post-DSS provided by a pathologist blinded to the groups and the study. Results representative of at least 3 independently performed experiments and displayed as mean ± SEM. *p < 0.05; ***p < 0.001; ****p < 0.0001 (non-parametric test followed by a Mann–Whitney test; one/two-way ANOVA analysis followed by a Tukey/Bonferroni *post hoc* test for three group analysis).

Dextran sodium sulfate (DSS)-induced colitis is a model commonly used to study host–microbial interactions characterized by a defect in mucus gel layer and microbiota alterations (17). In addition, the administration of DSS disrupts the intestinal barrier and effectively mimics the clinical and histological features of IBD patients (49). Therefore, we next investigated whether Atg16L1^T300A/T300A^ mice will be more sensitive to DSS-induced colitis. Accordingly, WT and Atg16L1^T300A/T300A^ mice were administrated with 1.5% DSS in drinking water for 7 days followed by 3 days of regular drinking water, and then the extent of colitis was evaluated. We observed that the weight loss in Atg16L1^T300A/T300A^ mice was comparable with WT mice (**Figure 5A**). However, the colon length of Atg16L1^T300A/T300A^ mice was shorter than that of WT mice (**Figures 5B, C** **)**. In addition, the colon section was obtained and H&E staining was performed. We found that Atg16L1^T300A/T300A^ mice exhibited more intense inflammation in the colon section compared than did WT mice, as indicated by blinded assessment of histopathologic scores (**Figures 5D, E** **)**. Overall, our data indicated that the presence of Atg16L1 T300A resulted in defects in mucin granule exocytosis and impaired mucus layer formation that altered the microbial community in Atg16L1^T300A/T300A^ mice. Because there are many other defects in Atg16L1^T300A/T300A^ mice, the dysbiosis observed in Atg16L1^T300A/T300A^ mice is probably one of the important factors that contributed to the Atg16L1^T300A/T300A^ variant’s susceptibility to Crohn’s disease.

## Discussion

The role of the gut microbiota in IBD pathogenesis is well established, but whether genetic risk loci directly affect specific gut microbial population frequency is not known. Here we showed the presence of the CD risk allele Atg16L1 T300A in mice regulated gut microbiota. Atg16L1^T300A/T300A^ mouse intestine mucus displayed an IBD-associated species even under a steady state, which may be one of the important factors that lead to the Atg16L1 T300A carriers being more sensitive to IBD. Mechanically, diminished mucin secretion of Atg16L1^T300A/T300A^ goblet cell resulted in microbiota dysbiosis in Atg16L1^T300A/T300A^ mice.

To derive a comprehensive definition of microbial dysbiosis relevant to IBD, we analyzed the microbiome from the feces and mucus of the gastrointestinal tract and found that the diversity of bacteria is not uniformly distributed between WT and Atg16L1^T300A/T300A^ mice. For example, *Ruminococcaceae* were increased in Atg16L1^T300A/T300A^ mice in both feces and mucosa, but *Tyzzerella* and *Mucispirillum* were increased and *Akkermansia* was reduced only in the intestinal mucosa of Atg16L1^T300A/T300A^ mice. Our results here support an understanding of the gastrointestinal regions as microenvironments where the properties of each environment drive the taxonomic composition (50).

The homeostasis of the gut microbiota is essential for maintaining intestinal health, and clear evidence of microbial dysbiosis has been shown in patients with IBD (51, 52). Consistent with previous reports (19), our data showed that Atg16L1^T300A/T300A^ mice displayed an altered gut microbial composition at a steady state. More specifically, *Ruminococcaceae* were enriched in CD patients of small intestine mucosa and often co-occurring with increased disease activity (53, 54), which may also explain that the Atg16L1 T300A variant confers increased risk for the development of Crohn’s disease.

*Akkermansia*, as a mucin-degrading bacteria (38), could continuously reshape and refresh the mucus layer, thereby creating a healthy environment for epithelial cells and maintaining the integrity of the intestinal barrier (43). *In vitro*, Reunanen et al. (26) found that *Akkermansia* could strengthen the intestinal epithelial integrity and fortify a destroyed gut barrier. Our results showed that *Akkermansia* was reduced in both the colon and distal ileum mucosa of the Atg16L1^T300A/T300A^ mice, which was consistent with the reports showing that *Akkermansia* had been decreased in ulcerative colitis and Crohn’s disease patients than in healthy individuals (5, 31). In addition, another mucus-dwelling bacterium, *Mucispirillum schaedleri*, increased in both the colon and distal ileum mucosa of the Atg16L1^T300A/T300A^ mice, was also reported by Caruso et al., which can trigger Nod2^−/–^&Cybb^−/−^ (double–KO) mice developing spontaneous colitis (35). These results suggest that both *Akkermansia* and *Mucispirillum* may play a role in triggering colitis in T300A genetic susceptibility. Furthermore, there is evidence that mucus-consuming bacteria with increased prevalence in IBD patients are also better mucus utilizers *in vitro* (21), indicating an important role in mucus utilization, mucosal proximity, and disease.

Atg16L1 T300A SNP has multiple effects on the organism. Our results show that goblet cell dysfunction in Atg16L1^T300A/T300A^ mice resulted in intestinal microbiota disorder. In fact, the barrier integrity associated with defects in Paneth cell antimicrobial peptide secretion was also reduced in Atg16L1 hypomorphic mice (9) and Atg16L1^T300A/T300A^ mice (55). Furthermore, increased production of inflammatory cytokines by Atg16L1-deficient innate immune cells in response to infiltrating bacteria (56) can activate an adaptive immune response to the gut microbiota. Therefore, the occurrence of the T300A variant affecting IBD is caused by a variety of factors, and what is the nature of the co-occurrence relationships between these factors needs to be further studied.

IBD symptoms can vary in nature and severity as well as disease location. While some patients will respond to treatment, others will not, suggesting that subtypes of the disease may be dependent on many factors such as genetics, immunity, and the microbiota. Our study revealed that the risk allele Atg16L1 T300A, an SNP associated with an increased risk of CD, contributes to dysbiosis in mice, which was due to the dysfunction of goblet cells. These data shed light on the etiology of CD and provided a new perspective for the individualized treatment of IBD. Further clinical studies in humans with IBD need to be investigated.

## Data Availability Statement

The datasets presented in this study can be found in online repositories. The names of the repository/repositories and accession number(s) can be found in the following: https://www.ncbi.nlm.nih.gov/, PRJNA735432; PRJNA735442.

## Ethics Statement

The animal study was reviewed and approved by the Research Ethics Committee of the Institute of Microbiology, Chinese Academy of Sciences (IMCAS).

## Author Contributions

HL, PG, BJ, and NL performed the experiments. HL, PG, and FZ designed the study. HL, PG, and FZ analyzed the data. BZ provided expertise and reagents. PG and FZ supervised the study. HL, PG, and FZ wrote the manuscript. All authors contributed to the article and approved the submitted version.

## Funding

This work was supported by the National Natural Science Foundation of China grants (31870880 and 31470861 to FZ).

## Conflict of Interest

The authors declare that the research was conducted in the absence of any commercial or financial relationships that could be construed as a potential conflict of interest.

## Publisher’s Note

All claims expressed in this article are solely those of the authors and do not necessarily represent those of their affiliated organizations, or those of the publisher, the editors and the reviewers. Any product that may be evaluated in this article, or claim that may be made by its manufacturer, is not guaranteed or endorsed by the publisher.

## Supplementary Material

Supplementary Figure 1
**(A)** Beta-diversity of colonic mucosa microbiota profiles of WT and Atg16L1^T300A/T300A^ mice samples illustrated with PCoA using unweighted UniFrac distance metrics. Samples colored by different group: WT (green, n = 8); Atg16L1^T300A/T300A^ (red, n = 7). The proportions of bacterial taxa in the colon **(B)** and ileum **(C)** mucosa were significantly differentially represented in WT mice vs. Atg16L1^T300A/T300A^ mice. Statistically significant differences were then evaluated by Tukey or Wilcoxon rank-sum *post hoc* tests.*p < 0.05, **p < 0.01.
