# Supplementary figures and images for: IBD-Associated Atg16L1T300A Polymorphism Regulates Commensal Microbiota of the Intestine
Source: Front Immunol. 2022 Jan 27;12:772189. doi: 10.3389/fimmu.2021.772189 (PMC8829142; doi:10.3389/fimmu.2021.772189)

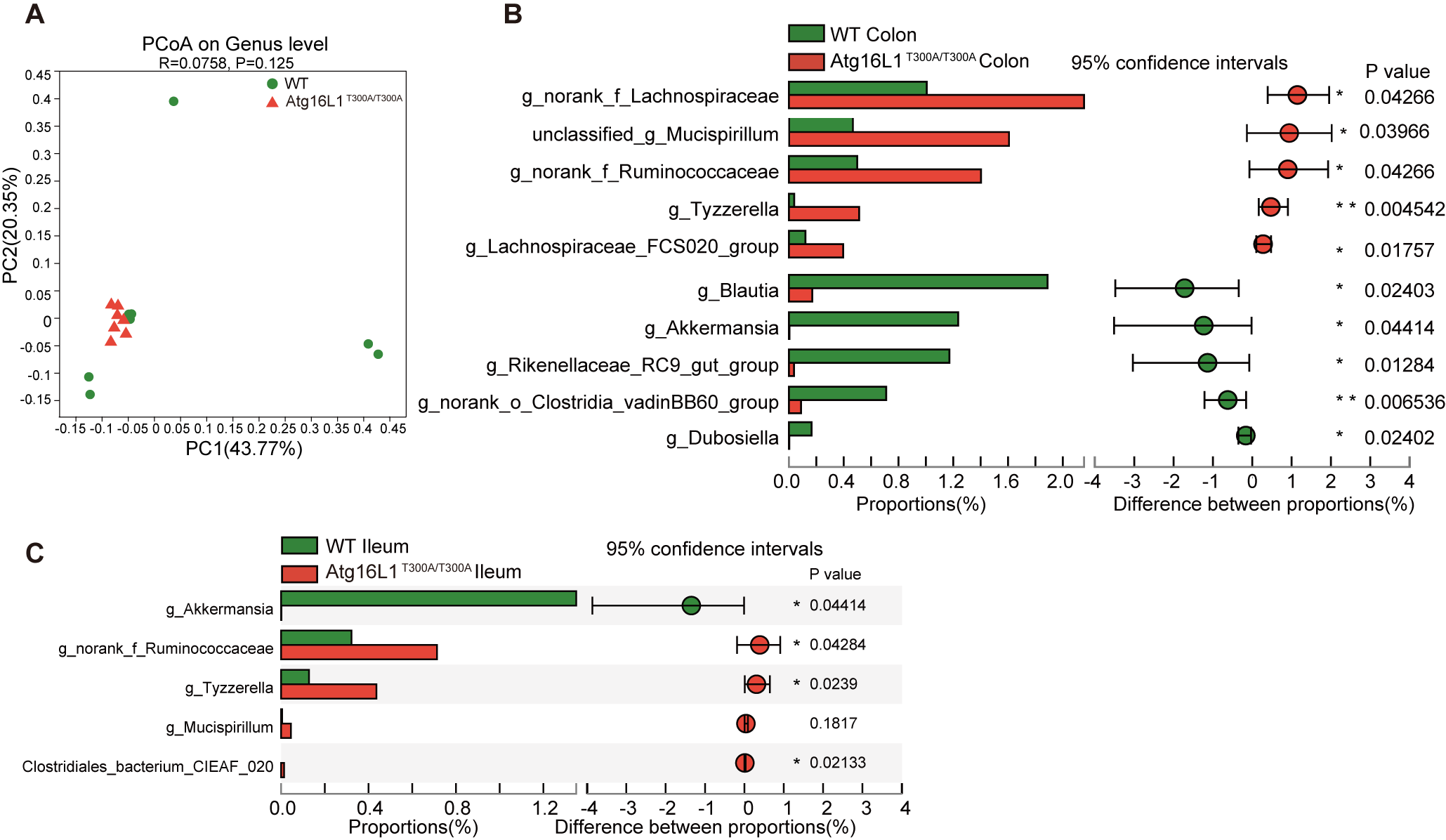

Supplement: Supplementary Figure 1 — (A) Beta-diversity of colonic mucosa microbiota profiles of WT and Atg16L1T300A/T300A mice samples illustrated with PCoA using unweighted UniFrac distance metrics. Samples colored by different group: WT (green, n = 8); Atg16L1T300A/T300A (red, n = 7). The proportions of bacterial taxa in the colon (B) and ileum (C) mucosa were significantly differentially represented in WT mice vs. Atg16L1T300A/T300A mice. Statistically significant differences were then evaluated by Tukey or Wilcoxon rank-sum post hoc tests.*p < 0.05, **p < 0.01. [file Image_1.tif]
